# Native Voices: Native Peoples’ Concepts of Health and Illness in New Mexico: opening a local conversation by hosting a national traveling exhibit

**DOI:** 10.5195/jmla.2017.257

**Published:** 2017-07-01

**Authors:** Patricia V. Bradley, Laura J. Hall, Gale G. Hannigan, Frederick B. Wood

## Abstract

**Background:**

The University of New Mexico Health Sciences Library and Informatics Center hosted the National Library of Medicine’s *Native Voices: Native Peoples’ Concepts of Health and Illness* traveling exhibit. The authors’ goal was to promote local interest in the *Native Voices* exhibit, with an emphasis on making the exhibit content and materials available to American Indian communities throughout rural New Mexico.

**Case Presentation:**

We convened a daylong summit to highlight the exhibit and encourage discussion among 30 American Indian community health educators. The summit prompted the compilation and distribution of descriptions of 23 community projects that promote health and wellness. We also took a scaled-down version of the exhibit to 4 rural college campuses around the state that serve significant Native American student populations. Approximately 140 students and faculty interacted with the exhibit materials, and all 4 sites incorporated the exhibit into curriculum activities.

**Conclusions:**

A hosted national exhibit developed into a multifaceted, funded project that engaged with American Indian communities. We demonstrated successful field deployment of a downsized, portable version of the full traveling exhibit to make meaningful connections with members of our outreach population.

## BACKGROUND

*Native Voices: Native Peoples’ Concepts of Health and Illness (Native Voices)* [1] is an outgrowth of the National Library of Medicine’s (NLM’s) American Indian health information outreach work, starting with Tribal Connections in 1998 to 2003 in the Pacific Northwest and Southwest [2, 3], which focused on improving Internet connectivity and providing health information training. The *Native Voices* traveling exhibit concept emerged from NLM consultations with tribes held in 2003 to 2008 at Ft. Yates, North Dakota; Anchorage, Alaska; Honolulu, Hawaii; Santa Fe, New Mexico; and Seattle, Washington. The exhibit features interviews with healers, community members, and elders from tribes across the United States, allowing visitors to hear people’s stories in their own words. Conceptually, *Native Voices*: explores the interconnectedness of wellness, illness, and cultural life for Native Americans, Alaska Natives, and Native Hawaiians. Visitors...discover how Native concepts of health and illness are closely tied to the concepts of community, spirit, and the land. [4]

Pilot-testing of the *Native Voices* exhibit began in 2013, which laid the groundwork for nationwide deployment of the exhibit to 104 sites over 4 years, in partnership with the American Library Association (ALA). The University of New Mexico (UNM) Health Sciences Library and Informatics Center (HSLIC) in Albuquerque, New Mexico, served as one of the test sites. The HSLIC has served as the state’s National Network of Libraries of Medicine Resource Library for more than 20 years and is 1 of 2 academic health sciences libraries in New Mexico. The HSLIC employs a full-time native and distance services librarian and has ongoing outreach efforts to American Indians, with services focused on health information delivery, health information training, and collection development consultation.

New Mexico is one of the most rural states in the nation, averaging 17 people per square mile compared with a nationwide average of 87.4 people per square mile [5]. Geographically, it is the fifth largest state in the United States. New Mexico is home to 22 federally recognized American Indian tribes—19 distinct Pueblo, Jicarilla Apache, Mescalero Apache, and Eastern Navajo Nation—and 10.4% of New Mexico’s population is American Indian [6]. These tribes are historically indigenous to the region, maintaining their native languages, cultures, and traditions. Therefore, it was important to take the exhibit outside of Albuquerque to those who might have found it meaningful but were unable to travel to Albuquerque.

## STUDY PURPOSE

Our goal was to promote local interest in the *Native Voices* exhibit, with an emphasis on making the exhibit content and materials available to American Indian communities throughout rural New Mexico. With further support from NLM, the authors sought to extend the impact of the *Native Voices* exhibit with outreach activities to American Indian communities.

## CASE PRESENTATION

### Hosting the exhibit and summit

The UNM HSLIC hosted the *Native Voices* exhibit on the UNM campus in Albuquerque April through October 2015. The exhibit arrived in 3 500-pound crates, containing 6 banners with printed exhibit text and images and 6 iPad listening stations. The *Native Voices* exhibit was displayed in the lobby of the education building. The tracking of building use showed more than 23,000 visits during the period of the exhibit. Gayle DinéChacon, a UNM professor of family and community medicine and founder of the UNM Center for Native American Health, who delivered the opening keynote address, said the exhibit “validates our history, our people, and their voices.” She stressed the importance of sovereignty, community, ceremony, tradition, family, and the environment to American Indian people as well as life balance and the right to health care.

After consultation with leaders in American Indian health and HSLIC faculty, coordinated by HSLIC’s native and distance services librarian, we convened a daylong summit to bring together American Indian health educators, health advocates, and community health workers to discuss concepts of health and wellness. The idea of the summit evolved from the *Native Voices’* subtitle, *Native People’s Concepts of Health and Illness.* We sought to highlight New Mexico American Indians’ concepts of health and wellness, in contrast to NLM’s concepts of health and illness.

Thirty members of the Apache, Navajo, and Pueblo communities traveled to Albuquerque on September 18, 2015, to attend the “Concepts of Health and Wellness among Native Peoples of New Mexico” summit. Activities included time to view the exhibit, a smudge ceremony (i.e., a purification ritual involving the burning of native plants), a morning panel session, networking opportunities, and an afternoon sharing circle of health and wellness projects from the community attendees. The topic of the morning panel discussion was “How do Native people in New Mexico define health and wellness?” Panelists included a western-trained Navajo physician, a Navajo traditional medicine man, a National Institutes of Mental Health research fellow, and a Pueblo health educator. A representative from NLM gave an overview of the exhibit, and a health promotion educator from the Indian Health Service led participants in brief exercises from the *Physical Activity Kit**,* an Indian Health Service initiative to increase physical activity among Alaska Natives and American Indians. Presentations throughout the day touched upon exhibit themes from a local perspective. We planned and coordinated this program with campus and community contacts. Attendees received recordings of selected parts of the summit.

### Taking the exhibit on the road

Given the rural nature of New Mexico, we felt it was important to take the exhibit to those who might find it meaningful, rather than expecting those individuals to travel to Albuquerque. Two HSLIC faculty members scheduled days to take the mobile parts of the exhibit to colleges serving American Indian populations around the state. We selected colleges with a high percentage of American Indian students and arranged the visits with the college librarians, setting up a date, time, and location when and where the most number of people might see the exhibit. We took the exhibit banners and iPads by car to college campuses in Gallup, Taos, Grants, and Farmington, which are 140, 133, 83, and 182 miles from Albuquerque, respectively. Each visit lasted a day and included opportunities to extend the conversation about concepts of health and wellness.

- Field Site #1: UNM–Gallup campus, September 10, 2015. The exhibit was displayed in the library and received approximately 20 visitors. Local faculty scheduled visits by students enrolled in a Navajo language class.
- Field Site #2: UNM–Taos campus, September 15, 2015. The exhibit was displayed in the education building and received approximately 25 visitors. Audio clips from the exhibit were presented in a “Holistic Health and Healing Arts” class, with a guided discussion about concepts of health and wellness.
- Field Site #3: New Mexico State University–Grants campus, September 16, 2015. The exhibit was displayed in a cafe and received approximately 25 visitors. Students enrolled in a biology class received extra credit for incorporating information from the exhibit into a class project.
- Field Site #4: San Juan College campus in Farmington, September 21, 2015. The exhibit was displayed in the library reading room and conference room and was visited by approximately 50 students, including students in a “Rural and Multi-Cultural Health” class from the UNM–San Juan Center. An estimated 25 students attended a panel discussion about the exhibit, which included an HSLIC faculty member, representatives from NLM, and San Juan College nursing and dental hygiene faculty members.

We solicited feedback for all of our outreach activities. Summit participants completed written evaluation forms and provided favorable feedback. Comments included:

I will take some of the ideas that people shared to use in my community. The inspiration from presenters will also help me to be more inspired about the work I do.

Just a reminder of why we are in the health field.

Personally the information absorbed through this summit informs me of what is already being done so I don’t have to reinvent the wheel. Professionally, I have been able to network and meet new connections and connect with more resources. Thank you.

Feedback from the site visits was also positive, and the onsite discussions were lively. A total of thirty-one evaluation forms were completed at the time of the visits. Comments about the exhibit included:

many of these ideas can cross career paths; be mindful of peoples’ belief systems; have that holistic awareness

we have to meet individuals where they’re at, it’s not a one-size fits all

everything affects your well-being

these are short interviews and they make you want more

When asked, “What does it mean to you to be healthy?,” people responded:

being well-balanced

ability to be able to adapt to your situations

an awareness

being happy, free of disease, having the physical, emotional and mental ability to do what you want

health doesn’t have to mean “free of disease” but having quality of life

approaching your aspects of life with creativity

open heart and open mind

### Compiling and disseminating an activity resource kit

The positive outcomes of these activities led to an offer of additional funding from NLM, which was used to compile and disseminate an activity resource kit to document current health and wellness projects in American Indian communities and to fund educational activities for American Indian youth who were interested in health careers. The idea for an activity resource kit emerged from notes from the summit’s afternoon sharing session about community projects in New Mexico that improve health and wellness. The goal of the kit was to provide models for others to adapt and implement in their communities.

To solicit submissions for the activity resource kit, we created an input form with instructions for describing community projects using a standardized format that included date, community, audience, project goals, and activities. We sent the form by email and followed up with phone calls to those who participated in the summit. We also publicized the opportunity in the *Native Health Initiative* weekly newsletter [7], and the HSLIC’s native and distance services librarian traveled to native communities to promote the project. Specifically, she participated in the Pueblo of Laguna Sports and Wellness Diabetes Program, Men’s Health and Wellness Expo; she was invited to the Navajo Department of Health Suicide Prevention Initiative interdisciplinary meeting; she met with Mescalero Apache Tribe Community Health Representatives; and she participated in the Native American Libraries Special Interest Group meeting. To compile the activity resource kit, we hired a student worker who organized the project descriptions and images and designed the front cover. Contributors received $150 per submission and a printed copy of the kit.

The final published activity resource kit contains descriptions of 23 projects from across New Mexico. Projects in the kit describe health careers mentoring, mindfulness and stress management, tobacco awareness, fatherhood or parenting, and traditional approaches to wellness. As an example project, the goal of the Pueblo of Laguna Fatherhood Program is to strengthen parenting skills by enabling fathers to be active and engaged in the physical, social, and emotional growth of their children by providing resources such as workshops, planned family outings, and classroom activities. A print copy of the kit is available in the HSLIC. The bibliographic record and an electronic version was featured as a new resource in the Native Health Database. In addition, an electronic copy of the kit resides in the UNM institutional repository [8] and is discoverable via Google. To date, the kit has been downloaded more than 200 times, and it is currently being promoted in local high schools to provide models for developing community health projects.

Finally, in an effort to involve college-age students, we used NLM funding to offer scholarships for American Indian youth to attend a health-related conference of their choosing to encourage interest in health careers. We received four inquires; however, only one student was able to take advantage of the opportunity. Feedback indicated that it was difficult for students to take time off during an academic semester, and we experienced conflicts with university policies related to administrative and reimbursement guidelines.

## DISCUSSION

Librarians engage in outreach for many reasons and in many ways, with the common goal being to provide services beyond the walls of the physical library to constituents who may not be aware of what the library has to offer. Another goal is to reach out to and engage rural, minority, and underserved populations, with Native Americans in New Mexico frequently fitting this description. NLM has embedded these goals in its strategic planning since the late 1980s [9, 10].

Previous literature has described using exhibits to extend the library’s outreach. For example, in a compilation of examples of outreach as community engagement, Tucker discusses the value of hosting traveling exhibits as a way to build new and strengthen existing community partnerships [11]. Mitchell and Zwemer describe placing components of an ALA-Smithsonian Institution traveling exhibit, *A More Perfect Union: Japanese Americans and the U.S. Constitution,* and related materials in different locations around the library to increase exposure and invite discussion. They conclude that hosting traveling exhibits provides libraries with an opportunity to engage users and community members in meaningful conversations [12]. In a survey of libraries that hosted an NLM exhibit, twenty-six of forty respondents indicated that they hosted other activities alongside the exhibit, such as special speakers, panel discussions, and film viewings [13]. However, traveling into the community with a scaled-down version of the exhibit was not discussed.

We found that taking elements of the exhibit to locations around the state illustrated how *Native Voices* could stimulate local tribal and community involvement. A barrier to local use and display of *Native Voices* and other traveling exhibits is cost. However, the scaled-down version of the exhibit described in this project reduced costs substantially by eliminating shipping costs and using only iPads and banners, but not heavy stands. Additionally, *Native Voices informational materials*, including a poster, are available for free download and can be printed locally.

Through our outreach efforts in hosting *Native Voices,* we developed a multifaceted approach to foster meaningful conversations in the rural communities of New Mexico, creating a dialogue with young students, community health care workers, and community leaders about the meaning of health and wellness. The *Native Voices* traveling exhibit informed these conversations by providing a catalyst to begin the exchange of ideas. The NLM exhibit gathered multiple viewpoints about health from across the country; likewise, we found various concepts and attitudes about health among New Mexico American Indian communities. These concepts are tied to the value systems of the individual and community. What emerged was representative of local voices versus the library or the exhibit as the “authoritative” source. Opening a dialogue with individuals during our one-day trips and the summit resulted in sharing and exchanging ideas. The activity resource kit extended the conversation beyond those who participated into native and rural communities of New Mexico. The outreach librarian was invited to participate in a campus health careers fair as a follow-up to the visit to the Grants field site #3. Furthermore, we are planning to provide each of the four tribal colleges in New Mexico with *Native Voices* iPads and educational materials using additional NLM support.

We learned several lessons from our experience. First, timing is important. It would have been better to coordinate site visits with faculty a semester in advance so that the content could be better integrated into the curriculum. We also believe that the visits could have been more coordinated with campus activities if they had been scheduled mid-semester, while students were working on class projects.

Second, involving local librarians is critical. We found local librarian involvement to be key to our success because of their familiarity with local faculty and curricula and their ability to make local arrangements.

Third, for successful outreach, relationships are everything. Developing the invitee list for the summit and deciding on the locations of the site visits depended on contacts, reputation, and professional networks. This type of outreach would not be possible without long-term trust and goodwill, especially in communities where there is a history of mistrust.

Finally, know your strengths, and when given an opportunity, take advantage of it. Ideally, we would have preferred more control over timing and administrative deadlines. However, we were flexible and depended on our established networks to accomplish the goal of extending the exhibit’s impact to local communities.

Hosting NLM’s *Native Voices* national exhibit at our institution created subsequent opportunities for the library to engage in local and regional outreach to Native American communities. Although outreach efforts can be time consuming to plan and manage, hosting a traveling exhibit can provide libraries great opportunities for meaningful activities and engagement with the community.
